# Rosmarinic acid improves hypertension and skeletal muscle glucose transport in angiotensin II-treated rats

**DOI:** 10.1186/s12906-019-2579-4

**Published:** 2019-07-08

**Authors:** Mujalin Prasannarong, Vitoon Saengsirisuwan, Juthamard Surapongchai, Jariya Buniam, Natsasi Chukijrungroat, Yupaporn Rattanavichit

**Affiliations:** 1 Department of Physical Therapy, Faculty of Associated Medical Sciences, Chiang Mai University, Chiang Mai, 50200 Thailand; 2 Exercise Physiology Laboratory, Department of Physiology, Faculty of Science, Mahidol University, Bangkok, 10400 Thailand; 3 Faculty of Physical Therapy, Mahidol University, Nakhonpathom, 73170 Thailand; 4 Faculty of Physical Therapy, Huachiew Chalermprakiet University, Samut Prakan, 10540 Thailand; 5 Division of Physical Therapy, Faculty of Physical Therapy, Srinakharinwirot University, Nakhon Nayok, 26120 Thailand

**Keywords:** Rosmarinic acid, Angiotensin II, Skeletal muscle, Insulin resistance, Extracellular signal-regulated kinase, Mitogen-activated protein kinase

## Abstract

**Background:**

Rosmarinic acid (RA) is a natural pure compound from herbs belonging to the *Lamiaceae* family, such as rosemary, sage, basil, and mint. The antioxidant, angiotensin-converting enzyme inhibitory, and vasodilatory effects of RA have been revealed. Angiotensin II (ANG II) is a potent agent that generates hypertension and oxidative stress. Hypertension and skeletal muscle insulin resistance are strongly related. The aim of this study was to evaluate the effects of acute and chronic RA treatment on blood pressure and skeletal muscle glucose transport in ANG II-induced hypertensive rats.

**Methods:**

Eight-week-old male Sprague Dawley rats were separated into SHAM and ANG II-infused (250 ng/kg/min) groups. ANG II rats were treated with or without acute or chronic RA at 10, 20, or 40 mg/kg. At the end of the experiment, body weight, liver and heart weights, oral glucose tolerance, skeletal muscle glucose transport activity, and signaling proteins were evaluated.

**Results:**

Both acute and chronic RA treatment decreased systolic, diastolic, and mean arterial blood pressure. Only acute RA at 40 mg/kg resulted in a reduction of fasting plasma glucose levels and an induction of skeletal muscle glucose transport activity. These effects might involve increased ERK activity in skeletal muscle. Meanwhile, chronic RA treatment with 10, 20, and 40 mg/kg prevented ANG II-induced hyperglycemia.

**Conclusions:**

Both acute and chronic RA treatment attenuated ANG II-induced cardiometabolic abnormalities in rats. Therefore, RA would be an alternative strategy for improving skeletal muscle glucose transport and protecting against ANG II-induced hypertension and hyperglycemia.

## Background

Rosmarinic acid (RA) is a natural pure compound from herbs that belong to the *Lamiaceae* family, such as rosemary, sage, basil, and mint. These plants are widely and routinely used in cooking recipes. Rosmarinic acid is an ester of caffeic acid and 3,4-dihydroxyphenyllactic acid. The biological benefits of chronic use of RA on cardiometabolic abnormalities have been revealed. Rosmarinic acid reduces blood pressure by its angiotensin-converting enzyme (ACE) inhibitory effects [1], promotes nitric oxide production, and downregulates endothelin-1 (ET-1) production [2]. Chronic treatment with RA improves whole-body insulin sensitivity in fructose-fed hypertensive rats [2] and high-fat diet (HFD)-induced diabetic rats [3, 4]. It also reversed streptozocin-induced decreases in skeletal muscle plasma membrane GLUT-4 content in diabetic rats [4]. However, the mechanisms through which RA increases glucose uptake need to be elucidated.

Angiotensin II (ANG II) is a potent hypertensive agent. It is involved in the generation of reactive oxygen species (ROS) that activate p38 MAPK, decrease Akt phosphorylation, and decrease GLUT-4 translocation in skeletal muscles [5–7]. The antioxidant properties of RA inhibit the production of ROS via c-Jun N-terminal kinase (JNK) and extracellular signal-regulated kinase (ERK) in a cell death model of cardiac muscle [8]. A previous study reported that ERK plays a crucial role in the therapeutic actions of RA in the hippocampus [9]. Moreover, exercise and 5-aminoimidazole-4-carboxamide-1-beta-d-riboside (AICAR) increase skeletal muscle glucose transport through the activation of ERK and adenosine monophosphate-activated protein kinase (AMPK) activities [10]. Together, RA might induce skeletal muscle glucose transport via the ERK pathway. In addition, RA could improve both cardiovascular and metabolic problems in hypertensive conditions. Therefore, the aim of this study was to evaluate the effects of acute and chronic RA administration on blood pressure and skeletal muscle glucose transport in rats treated with ANG II. Moreover, this study evaluated the signaling pathways involved in skeletal muscle glucose transport.

## Methods

### Chemicals

Rosmarinic acid was purchased from Sigma–Aldrich Inc. (St. Louis, MO). Angiotensin II was purchased from AnaSpec Inc. (Fremont, CA). Rat insulin radioimmunoassay (RIA) kits were purchased from Millipore (St. Charles, MO). Glucose enzymatic colorimetric tests were purchased from HUMAN Gesellschaft fÜr Biochemica und Diagnostica mbH (Wiesbaden, Germany). 2-[1,2-^3^H] deoxyglucose and [U-^14^C] mannitol were purchased from PerkinElmer Life Sciences (Boston, MA). Antibodies were purchased from Cell Signaling Technology Inc. (Beverly, MA).

### Animals

Experiments were carried out using 8-week-old male Sprague Dawley rats weighing 260–290 g from the National Laboratory Animal Center, Nakhon Pathom, Thailand. All rats were housed in a strict hygienic conventional housing system. Each rat was placed in a 9 × 12 × 6 in. cage with corn cob bedding at the Center of Animal Facilities, Faculty of Science, Mahidol University. The room temperature was controlled at 22 °C with a 12:12-h light-dark cycle (light on from 0600 to 1800 h). Rats had free access to water and pellet rat chow (Perfect Companion, Samutprakarn, Thailand). One week after arrival, rats were randomly assigned into the SHAM (control groups, *n* = 10 rats/group) and ANG II-treated groups (experimental groups, *n* = 10 rats/group). The sample size was calculated from blood pressure data according to Karthik et al., 2011 [2] by using Minitab 14 (Minitab Inc., State College, PA). ANG II (250 ng/kg/min) was subcutaneously delivered for 14 days by implanting a mini-osmotic pump (model 2002, DURECT Corporation, Cupertino, CA) on the back and slightly posterior to the scapulae. To study the acute effects of RA, 14-day ANG II-treated rats received a single dose of 10, 20, or 40 mg/kg RA by a single gavage. A pharmacokinetic study of RA had reported that the t_1/2_ of RA was 63.9 min [11]. The distribution of RA in skeletal muscle tissue had been observed 30 min after a single gavage [12]. Therefore, blood and tissue were collected 30 min after a single gavage, and the concentration of RA in blood and tissues was expected to be high. To assess the chronic effects of RA and to minimize the acute effects of RA, blood and tissues were collected at least 16 h after the most recent treatment. This study design was previously used in our study to evaluate the chronic effects of *Curcuma comosa* Roxb. on whole-body and skeletal muscle insulin sensitivity [13]. Rats in the SHAM and ANG II groups were gavaged with water and considered controls. In a separate study, the chronic effects of RA were assessed in rats that received 10, 20, or 40 mg/kg RA by gavage at 1600–1700 h for 14 consecutive days. Blood pressure was measured weekly by a tail cuff plethysmography apparatus using the Coda Monitoring system (Kent Scientific Corporation, Torrington, CT). Blood and tissue collections were performed at 0900–1200 h. Before tissue collection, rats were deeply anesthetized by intraperitoneal injection of thiopental (100 mg/kg). Respiratory rate, responses to noxious stimuli, and spontaneous responses were observed throughout the collection. After muscle dissection, other tissues were collected, and the rats were sacrificed by removal of the heart.

### Oral glucose tolerance test (OGTT)

Glucose tolerance tests were performed to determine the whole-body insulin sensitivity. In the evening (1800 h) on the day before the test, rats were restricted to 4 g of chow. In the next morning (0800–0900 h), rats were gavaged one time with 1 g/kg of glucose. Tail blood was collected into microfuge tubes containing anticoagulant (18 mM final concentration of EDTA) before and 15, 30, 60, and 120 min after the glucose feeding (1 g/kg). The blood samples were centrifuged at 13000×g at 4 °C for 1 min. Then, plasma samples were collected to determine glucose and insulin concentrations [14]. After the test, each rat was given sterile 0.9% saline subcutaneously as soon as possible for the replacement of the body fluid loss. Furthermore, plasma insulin and glucose concentrations were measured by RIA and enzymatic colorimetric tests, respectively.

### Glucose transport activity (GT)

Forty-eight hr after performing the OGTT, rats were restricted to 4 g of chow at 1800 h. Each rat was weighed and deeply anesthetized with an intraperitoneal injection of thiopental (100 mg/kg) before a dissection of soleus muscle. Then, soleus muscle was subsequently divided into two strips. Each muscle strip (~ 25 mg) was incubated at 37 °C for 60 min in 3 ml of oxygenated Krebs–Henseleit buffer (KHB) supplemented with 8 mM D-glucose, 32 mM D-mannitol, 0.1% radioimmunoassay-grade bovine serum albumin, and the presence or absence of 2 mU/ml insulin. After incubation, the muscle strips were rinsed at 37 °C for 10 min in 3 ml of oxygenated Krebs–Henseleit buffer (KHB) containing 40 mM mannitol and insulin, if previously present. Finally, the muscle strips were incubated for 20 min in 2 mL of KHB containing 1 mmol/L 2-[1,2-^3^H] deoxyglucose (2-DG (300 μCi/mmol), 39 mmol/L [U-^14^C] mannitol (0.8 μCi/mmol), 0.1% BSA, and insulin, if previously present. Each flask was gassed with 95% O_2_–5% CO_2_ throughout the incubation period of the experiment. At the end of the incubation, the muscle strips were removed from the flasks, had the excess fat and connective tissue trimmed off, were frozen with liquid nitrogen, and immediately weighed. Then, the muscle strips were solubilized in 0.5 ml of 0.5 N NaOH for 1 h and mixed with 10 ml of a scintillation cocktail. The specific intracellular accumulation of 2–DG was determined by subtracting the ^3^H activity in the extracellular space from the total ^3^H activity in each muscle strip [15]. The specific intracellular accumulation of 2–DG was determined using mannitol to correct for the extracellular accumulation of 2–DG. Glucose transport activities were measured as the intracellular accumulation of 2–DG (in pmol/mg muscle wet weight/20 min) [15].

### Skeletal muscle protein abundance and phosphorylation using immunoblotting

The soleus muscle from the other leg was dissected and subsequently divided into two strips. The muscle strips were incubated in the same solution type that was used for measuring GT in the presence or absence of 2 mU/ml insulin. After incubation, each muscle strip was trimmed of excess fat and connective tissue, quickly frozen in liquid nitrogen and kept at − 80 °C until performing immunoblotting. The muscle strips were homogenized in ice-cold lysis buffer: 50 mM HEPES (pH 7.4), 150 mM NaCl, 1 mM CaCl_2_, 1 mM MgCl_2_, 2 mM EDTA, 10 mM NaF, 20 mM sodium pyrophosphate, 20 mM β-glycerophosphate, 10% glycerol, 1% Triton X-100, 2 mM Na_3_VO_4_, 10 μg/ml aprotinin and leupeptin, and 2 mM PMSF. After a 20-min incubation on ice, the homogenates were centrifuged at 13000×g for 20 min at 4 °C. Proteins in the homogenate were separated on polyacrylamide gel and transferred electrophoretically onto nitrocellulose paper. The blots were incubated with an appropriate dilution of commercially available antibodies (Cell Signaling Technology Inc., Beverly, MA) against phospho-Akt (Ser473) (#9271; 1:800), Akt (#9272; 1:800), phospho-GSK-3α/β (Ser21/9) (#9331S; 1:1000), GSK-3α/β (#5676S; 1:1000), phospho-ERK1/2 (Thr202/Tyr204) (#4377; 1:1000), ERK1/2 (#4695; 1:1000), phospho-p38 MAPK (Thr180/Tyr182) (#9211; 1:800), p38 MAPK (#9212; 1:800), phospho-SAPK/JNK (Thr183/Tyr185) (#9251; 1:800), SAPK/JNK (#9252; 1:1000), and GAPDH (#2188; 1:3000). Subsequently, all blots were incubated with anti-rabbit IgG HRP-linked antibody (#7074; 1:1500). Protein bands were visualized by enhanced chemiluminescence. Images were digitized on a C-Digit Blot Scanner (LI- COR Biotechnology, Lincoln, NE), and band intensities were quantified using Image Studio Software version 3.1.

### Statistical analysis

The values of the collected data were reported as the means ± SE. One-way analyses of variance (ANOVA) with Fisher’s Least Significant Difference (LSD) post hoc tests were used to determine significant differences among the groups. Statistical analyses were performed using SPSS 17.0 (SPSS Inc., Chicago, IL). The significance level of the study was considered a *P* value < 0.05.

## Results

### Effects of ANG II on blood pressure, body weight, and organ weights

**Fig. 1:**
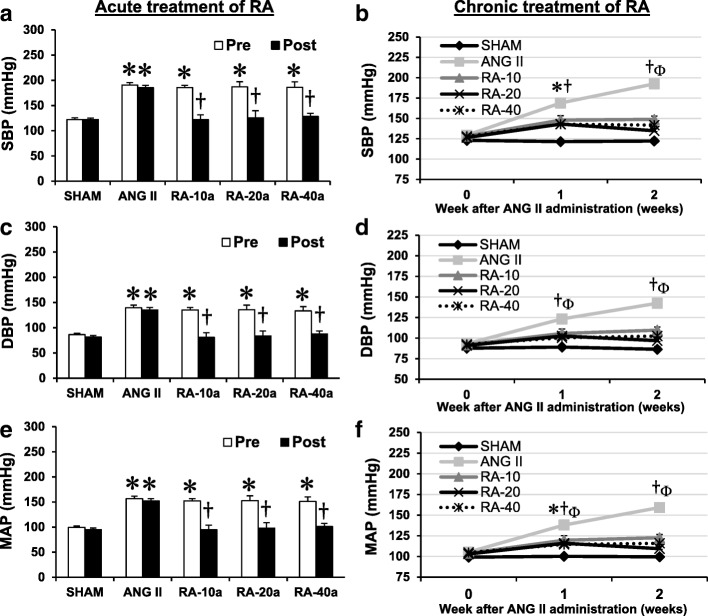
Systolic blood pressure (SBP), diastolic blood pressure (DBP), and mean arterial blood pressure (MAP) in SHAM, ANG II, acute RA treatment (RA-10a, -20a, and -40a mg/kg) (**a**, **c**, **e**), and chronic RA treatment (RA-10c, -20c, and -40c mg/kg) (**b**, **d**, **f**) groups. Values are the mean ± SE. *P < 0.05 vs SHAM group; ^†^*P* < 0.05 vs ANG II group; Φ*P* < 0.05, R-10c vs SHAM group

After administration of ANG II for 14 days, systolic, diastolic, and mean arterial blood pressure increased approximately 30–40 mmHg relative to the first week after ANG II administration. At the end of the study, ANG II increased blood pressure levels by 49–63 mmHg (Fig. 1, *P* < 0.05). The final body weights of the ANG II rats were significantly reduced compared with the SHAM rats (Table 1 and Table 2). At the end of the experiment, the liver weight to body weight ratio was not significantly changed, whereas the heart weight to body weight ratio increased by 0.77–0.95 g/kg (Table 1 and Table 2; *P* < 0.05).

**Table 1:** Animal characteristics and glycemic control in SHAM and ANG II-treated rats and in ANG II-treated rats following acute administration of RA at 10, 20, or 40 mg/kg

	SHAM			ANG II				RA-10a				RA-20a				RA-40a			
Body weight (g)																			
Initial weight	373.55	±	5.58	372.25	±	5.83		364.41	±	9.07		365.47	±	7.23		368.03	±	5.48	
Final weight (BW)	408.53	±	6.34	366.40	±	13.23	*	358.02	±	9.77	*	362.86	±	9.28	*	358.94	±	9.18	*
Liver weight (LW; g)	10.80	±	0.31	9.30	±	0.20	*	9.25	±	0.25	*	9.66	±	0.50	*	9.93	±	0.50	*
LW/kg BW (g/kg)	26.96	±	0.85	27.04	±	0.86		26.67	±	0.58		27.86	±	0.82		28.01	±	0.85	
Heart weight (HW; g)	1.12	±	0.04	1.26	±	0.02		1.27	±	0.04		1.29	±	0.04		1.31	±	0.03	
HW/kg BW (g/kg)	2.81	±	0.07	3.67	±	0.17	*	3.66	±	0.11	*	3.75	±	0.13	*	3.71	±	0.12	*
Fasting plasma glucose (mmol/l)	6.81	±	0.07	8.35	±	0.45	*§	8.29	±	0.25	*§	8.33	±	0.38	*§	7.18	±	0.32	
Fasting plasma insulin (mU/l)	39.74	±	2.67	35.46	±	6.61		28.03	±	3.83		34.37	±	4.51		33.23	±	4.80	
HOMA-IR	11.96	±	1.18	13.10	±	3.14		10.62	±	1.49		12.99	±	2.24		10.40	±	1.83	
Glucose AUC (mg/ml/min*10^4^)	1.85	±	0.04	2.19	±	0.15		2.32	±	0.22		2.46	±	0.18		2.34	±	0.13	
Insulin AUC (μU/ml/min*10^3^)	7.32	±	0.66	5.31	±	0.47	*	4.48	±	0.68	*	5.08	±	0.67	*	4.78	±	0.51	*
G-I index (μU/ml/min*mg/ml/min*10^7^)	13.90	±	1.40	12.33	±	1.70		9.77	±	1.98		11.71	±	1.67		10.66	±	1.22	

*P < 0.05 vs. SHAM group; § *P* < 0.05 vs. RA-40a group. HOMA-IR: homeostasis model assessment-estimated insulin resistance; G-I index: glucose-insulin index

**Table 2:** Animal characteristics and glycemic control in SHAM and ANG II-treated rats and in ANG II-treated rats following chronic administration of RA at 10, 20, or 40 mg/kg

	SHAM			ANG II				RA-10c				RA-20c				RA-40c			
Body weight (g)																			
Initial weight	374.10	±	6.08	372.92	±	7.30		383.71	±	5.87		373.28	±	5.89		379.00	±	4.71	
Final weight (BW)	400.80	±	4.79	369.16	±	9.57	*	363.99	±	11.71	*	383.77	±	11.85		373.24	±	9.82	
Liver weight (LW; g)	11.64	±	0.36	10.02	±	0.27	*	10.44	±	0.45		10.99	±	0.43		10.68	±	0.38	
LW/kg BW (g/kg)	28.80	±	0.75	28.53	±	0.64		28.63	±	0.60		28.64	±	0.69		28.58	±	0.55	
Heart weight (HW; g)	1.21	±	0.04	1.34	±	0.04		1.32	±	0.04		1.30	±	0.04		1.31	±	0.03	
HW/kg BW (g/kg)	2.98	±	0.10	3.75	±	0.14	*	3.65	±	0.14	*	3.42	±	0.13		3.52	±	0.11	*
Fasting plasma glucose (mmol/l)	6.83	±	0.06	8.12	±	0.36	*	7.18	±	0.15	†	7.10	±	0.18	†	7.08	±	0.16	†
Fasting plasma insulin (mU/l)	37.83	±	2.96	33.09	±	6.19		32.16	±	3.21		38.27	±	5.41		36.52	±	7.06	
HOMA-IR	12.24	±	1.35	14.27	±	3.44		10.50	±	0.98		12.87	±	2.14		12.53	±	2.64	
Glucose AUC (mg/ml/min*10^4^)	1.80	±	0.03	2.04	±	0.14		2.05	±	0.12		1.82	±	0.05		2.10	±	0.17	
Insulin AUC (μU/ml/min*10^3^)	7.08	±	0.71	5.46	±	0.61	*	5.52	±	0.70	*	5.17	±	0.53	*	5.26	±	0.66	*
G-I index (μU/ml/min*mg/ml/min*10^7^)	13.10	±	1.55	11.75	±	2.19		10.48	±	1.59		9.55	±	0.99		10.38	±	1.34	

* *P* < 0.05 vs. SHAM group; ^†^ *P* < 0.05 vs. ANG II group. HOMA-IR: homeostasis model assessment-estimated insulin resistance; G-I index: glucose-insulin index

### Effects of ANG II on whole-body and skeletal muscle insulin sensitivity

**Fig. 2:**
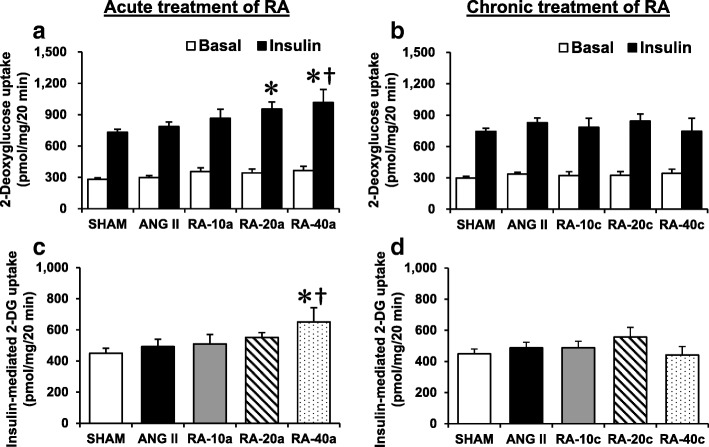
Glucose transport activity in basal and insulin-stimulated conditions, and differential changes among the basal and insulin-stimulated conditions (insulin-mediated 2-DG uptake) after SHAM, ANG II, acute RA (RA-10a, -20a, and -40a mg/kg) (**a**, **c**), and chronic RA (RA-10c, -20c, and -40c mg/kg) (**b**, **d**) treatment. Values are the mean ± SE. *P < 0.05 vs SHAM group; ^†^*P* < 0.05 vs ANG II group

**Fig. 3:**
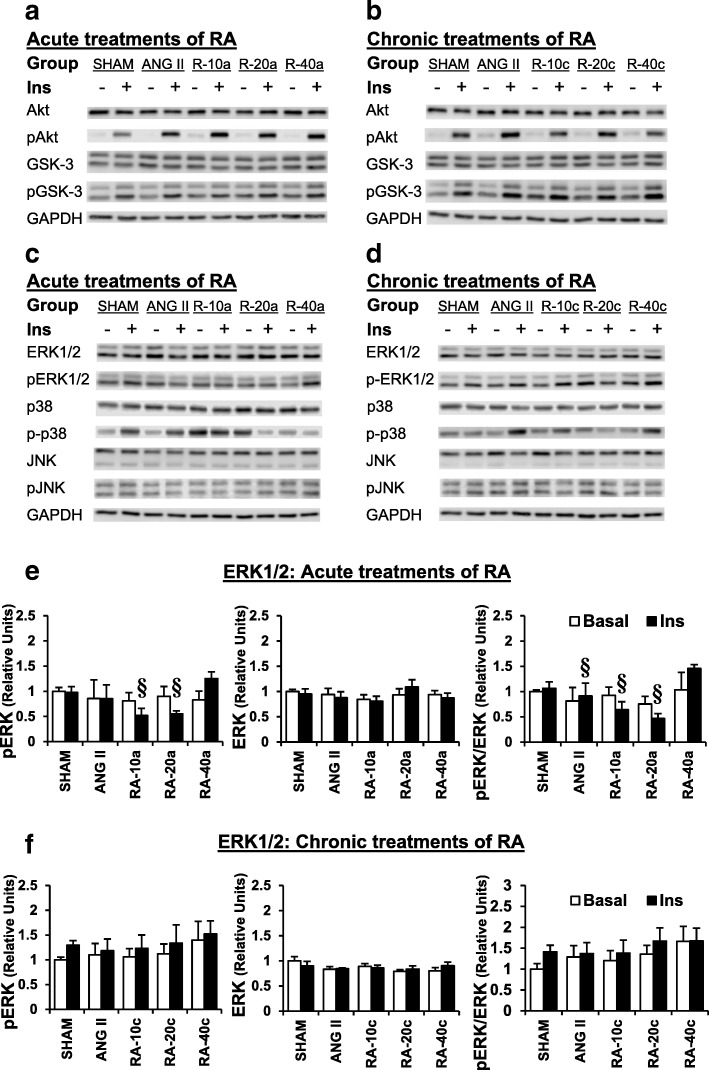
Western blots of insulin signaling and MAPK signaling after SHAM, ANG II, acute RA (RA-10a, -20a, and -40a mg/kg) (**a**, **c**), and chronic RA (RA-10c, -20c, and -40c mg/kg) (**b**, **d**) treatment. ERK1/2 phosphorylation, ERK, and ERK activity after SHAM, ANG II, acute RA (RA-10a, -20a, and -40a mg/kg) (**e**), and chronic RA (RA-10c, -20c, and -40c mg/kg) (**f**) treatment. Values are the mean ± SE. §*P* < 0.05 vs RA-40a group

Chronic infusion of ANG II increased fasting plasma glucose (1.29 and 1.54 mmol/l) and decreased insulin AUC (1.62 and 2.00 μU/ml/min*10^3^) levels when compared to SHAM conditions (Table 1 and Table 2; *P* < 0.05). However, there was no significant change in whole-body insulin sensitivity, including the homeostasis model assessment-estimated insulin resistance (HOMA-IR) and the glucose-insulin (G-I) index. Meanwhile, the study did not find any significant change from the ANG II infusion in slow-twitch muscle glucose transport activities (Fig. 2) and its protein elements (Fig. 3).

### Impact of acute and chronic RA on blood pressure and organ weights

All doses of acute and chronic RA treatment attenuated the blood pressure-increasing effects of ANG II. A reduction in blood pressure was found for all doses of acute RA treatment with means decreased by 46–64 mmHg, and for all chronic RA treatments, with means decreased by 33–58 mmHg (Fig. 1; *P* < 0.05). As shown in Table 1 and Table 2, liver weight to body weight ratios were not altered after RA treatment. Acute treatment with RA and chronic treatment with 10 mg/kg RA resulted in significantly increased heart weight to body weight ratios as was observed in the ANG II groups.

### Effects of RA treatment on whole-body and skeletal muscle insulin sensitivity

The fasting plasma glucose in the ANG II-treated rats was reduced by 1.17 mmol/l after a single gavage of 40 mg/kg RA. On the other hand, the fasting plasma glucose were decreased in the chronic RA treatment groups (10, 20, and 40 mg/kg) by 0.94–1.04 μU/ml/min*10^3^ (Table 1 and Table 2; *P* < 0.05). Neither acute nor chronic treatment with RA altered the HOMA-IR or G-I index. Interestingly, a single gavage administration of 20 and 40 mg/kg RA significantly increased insulin-stimulated glucose transport activity by 223 and 286 pmol/mg/20 min, respectively, compared with SHAM rats. However, only a single gavage of 40 mg/kg RA increased the insulin-mediated glucose transport activity (the difference between basal and insulin-stimulated glucose transport activity) by 201 pmol/mg/20 min, *P* < 0.05 (Fig. 2). Moreover, this study found increased ERK1/2 activity in insulin-stimulated conditions compared with the ANG II-treated group, *P* < 0.05 (Fig. 3).

## Discussion

This study evaluated the acute and chronic effects of RA in ANG II-induced hypertensive rats. The acute RA treatment decreased blood pressure and fasting plasma glucose and increased skeletal muscle glucose transport activity along with ERK activity. In addition, chronic RA treatment reduced blood pressure and fasting plasma glucose levels.

Systolic blood pressure-lowering effects of acute [16] and chronic [2, 17] RA treatments have been reported. These findings supported our results that acute and chronic treatment with RA reduced blood pressure, including systolic, diastolic, and mean arterial blood pressure in the SHAM rats (Fig. 1). The mechanisms involved in these effects included antioxidant [2, 8], ACE inhibition [1, 2, 16, 17], and vasodilation [2, 17] properties of RA. It increased nitric oxide (NO) and decreased ET-1 levels, ACE activity [1, 2], and angiotensin type 1 receptor (AT1R) expression [17] that consequently induced systemic vasodilation and consequently reduced the total peripheral resistance. Remarkably, the acute treatment with RA reduced blood pressure (46–64 mmHg; 33–42%) more than the chronic treatment (33–58 mmHg; 23–32%). This might involve a peak action of RA after acute administration (t_1/2_ of RA is 63.9 min [11]). Therefore, decreased blood pressure in the chronic RA-treated rats would simply be the result of the repeated effects of acute RA treatment.

This study is the first attempt to demonstrate an effect of a single oral administration of RA on skeletal muscle glucose transport. We found increased glucose transport activity and ERK activity. Previous studies have shown the effects of RA on muscle glucose transport activity and proposed mechanisms. Jayanthy et al. found increased skeletal muscle glucose transport in diabetic rats after chronic RA treatment [18]. They stated that this study finding was associated with decreased phosphorylation of IRS-1 (Ser307) and increased phosphorylation of AMPK, which facilitated GLUT-4 translocation to the plasma membrane. Vlavcheski et al. reported increased glucose transport in L6 rat muscle cells after a direct RA treatment that was partially dependent on AMPK but independent of PI3-K [19]. Similar to a study in B6 melanoma cells, RA had no effect on Akt and p38 phosphorylation [20]. The current study also found increased glucose transport activity (Fig. 2) without significant changes in Akt and p38 activity (Fig. 3). However, a previous paper reported that RA increased phosphorylation of p38 in the myocardial tissue of myocardial infarction rats [17]. In the present study, only increased ERK activity was observed. Stimulation of ERK can facilitate glucose transport in skeletal muscles and muscle cells [10, 21]. Atypical PKC (aPKC) activation of AMPK, ERK, and PDK1 are required for AICAR and metformin to facilitate skeletal muscle glucose transport, which is an insulin-independent pathway [10, 21]. Taken together, it is possible to state that increased ERK activity after a single RA gavage might lead to increased glucose transport activity in skeletal muscle. In addition to the insulin-dependent pathway, we suggest that a single gavage of 40 mg/kg RA may benefit skeletal muscle glucose transport through an alternate pathway.

Although the whole-body insulin sensitivity of ANG II-treated rats did not show a significant reduction during the oral glucose tolerance tests, significantly increased fasting plasma glucose and reduced insulin area under the curve were observed (Table 1 and Table 2). This would be a result of ANG II reducing beta cell function [22]. A unique finding of this study was that acute 40 mg/kg RA decreased fasting plasma glucose (Table 1). We also found a protective effect of chronic administration of 10, 20, and 40 mg/kg RA on ANG II-induced high levels of fasting plasma glucose (Table 2). Similar to our study, Govindaraj and Sorimuthu Pillai studied the effects of oral administration of RA (100 mg/kg) in diabetic rats for 30 days [3]. They reported that RA improved whole-body insulin sensitivity, preserved the beta cell mass of the pancreas, increased insulin levels, and decreased glucose levels. Karthik et al. reported improvements in systemic insulin sensitivity, blood pressure, lipid profile, myocardial damage markers, and oxidative stress markers in high fructose-fed rats treated with 10 mg/kg RA for 45 days [2]. In contrast, Mushtaq et al. reported no change in blood glucose levels in diabetic rats after 10 mg/kg RA treatment for 21 days [23]. Our results showed a protective effect of RA by reducing fasting plasma glucose. The acute lowering of the fasting plasma glucose in 40 mg/kg RA-treated rats may have been the result of RA-induced glucose transport activity (Fig. 2). Therefore, we suggest that both acute and chronic RA administration may be used in hypertensive and hyperglycemic models.

In the present study, acute and chronic RA had no effect on liver and heart weights (Table 1 and Table 2). This result was also confirmed by the first randomized controlled trial study in humans. They reported that a single dose of RA is safe for blood, kidney, and liver function [24]. However, there is no safety report following chronic treatment in humans. It is necessary to determine the mechanisms, dose, and treatment time of RA in future studies.

## Conclusion

Rosmarinic acid administration can attenuate ANG II-induced cardiometabolic abnormalities in rats. Acute RA treatment lowered blood pressure and fasting plasma glucose levels. Extracellular signal-regulated kinase (ERK) activity may be involved in increasing skeletal muscle glucose transport activity. Chronic RA treatment can prevent high blood pressure and hyperglycemia in hypertensive rats. Therefore, RA may be an alternative strategy for increasing skeletal muscle glucose transport and protecting against ANG II-induced hypertension and hyperglycemia.

## Data Availability

The datasets used and/or analyzed during the current study are available from the corresponding author on reasonable request.

## Abbreviations

ACE: Angiotensin converting enzyme
AMPK: Adenosine monophosphate-activated protein kinase
ANG II: Angiotensin II
ERK: Extracellular signal-regulated kinase
GAPDH: Glyceraldehyde-3-phosphate dehydrogenase
GLUT: Glucose transporter
GSK: Glycogen synthase kinase
MAPK: Mitogen-activated protein kinase
PI3-K: Phosphatidylinositol-4,5-bisphosphate 3-kinase
PKC: Protein kinase C
RA: Rosmarinic acid
ROS: Reactive oxygen species
SAPK/JNK: Stress-activated protein kinase/c-Jun N-terminal kinase
